# Correlation between ambient temperature and body weight of Hanwoo calves (Korean native cattle)

**DOI:** 10.5713/ab.24.0489

**Published:** 2025-02-27

**Authors:** Hyun Sang Kim, Jungeun Kim, Pilnam Seong, Won-Young Lee, Seongshin Lee, Jisoo Wi, Hye Ran Kim, Sung Dae Lee, Yookyung Lee

**Affiliations:** 1 Precision Animal Nutrition Division, National Institute of Animal Science, Rural Development Administration, Wanju, Korea; 2 Animal Resources Food Tech Division, National Institute of Animal Science, Rural Development Administration, Wanju, Korea; 3 Department of Beef & Dairy Science, Korea National College of Agricultures and Fisheries, Jeonju, Korea; 4 Smart Livestock Environment Division, National Institute of Animal Science, Rural Development Administration, Wanju, Korea

**Keywords:** Birth Season, Birth Weight, Carcass Weight, Hanwoo, Heat Stress

## Abstract

**Objective:**

Changes in ambient temperature negatively impact livestock productivity, with thermal stress causing physiological changes that affect beef quality and quantity. The calf stage is critical, as thermal stress during this period can have lasting effects on productivity. This study examined the impact of weather conditions on calf growth, carcass traits, and their interrelationships in Korean native Hanwoo steers.

**Methods:**

Data from 8,727 male Hanwoo calves were selected from 41,107 data points. Meteorological data were collected from 60 cities in Korea between 2016 and 2019. The input data included calf growth, carcass traits, and weather conditions at birth for each calf.

**Results:**

Temperature, relative humidity (RH), and temperature-humidity index (THI) increased from winter to summer and decreased from summer to winter. Summer-born calves had significantly lower birth body weight (BBW) than those born in winter. The average daily gain (ADG) of the calves was highest in winter and lowest in spring and fall. A high carcass weight (CW) was observed in steers born in summer and winter. BBW and ADG were negatively correlated with ambient temperature, RH, and THI. Positive relationships were observed between BBW, ADG, and CW. Only CW was positively correlated with the meat quality grade of steers. Thus, temperature negatively affects the BBW of male Hanwoo calves, in turn affecting the daily gain, CW, and meat quality grade.

**Conclusion:**

This study demonstrates that temperature, RH, and THI adversely affect the BBW of male Hanwoo calves, leading to reduced ADG and influencing CW and meat quality grade. Negative correlations were observed between BBW, ADG, and environmental factors, while CW showed positive correlations with BBW, ADG, and meat quality grade. These findings highlight the critical impact of climatic conditions on the growth performance and productivity of Hanwoo cattle.

## INTRODUCTION

The average temperature in Korea has increased by more than twice the global temperature over the past 100 years [1]. According to the Representative Concentration Pathway (RCP) 8.5 [2], there has been an observed increase in the average temperature and number of heat-wave days and tropical night days in Korea. Korea has distinct seasonal differences throughout the year due to its geographical characteristics; thus, there is a significant difference in the ambient temperature between winter and summer. Generally, cattle are housed in free barns in the Korean beef production system. These animals are subjected to alternating seasonal stress induced by cold temperatures in winter and extreme heat in summer [3].

Studies have been conducted to evaluate and predict the impact of climate change and abnormal weather on livestock caused by climate change and abnormal weather, as mandated by a law established in Korea. This stress influences both the quantity and quality of beef production through physiological alterations [4]. When animals undergo stress for various reasons, they exhibit decreased dry matter intake (DMI), which hampers animal growth and ultimately reduces productivity [5]. A previous study reported that heifers exhibit fewer fluctuations in body temperature with increasing ambient temperatures than lactating cows [6]. Calves and heifers radiate heat easily because they produce less metabolic energy and have a larger body area than their body weight (BW) [7]. Therefore, calves might be considered more thermostable than cows and steers; however, exposure to thermal stress during the calf stage can detrimentally affect future productivity. Heat-stressed calves demonstrate stunted growth [8,9] and increasing mortality [10]. Thus, the growth pattern of calves affected by weather is essential as a strategy to improve beef production.

Hanwoo, a native Korean cattle breed belonging to *Bos taurus* and is the dominant breed that accounts for 95.51% of domestic beef production. The thermoneutral zone of Hanwoo is presumed to be similar to that of other breeds [11]; however, with increasing summer temperatures frequently surpassing the upper critical temperature in summer in Korea, it is imperative to investigate the responses of Hanwoo to thermal stress. Studies have been conducted on Hanwoo calves [12,13] and steers [14,15] exposed to high temperatures to investigate their growth performance and physiological responses. However, knowledge regarding the relationships between climatic conditions and calf birth, growth rate, and subsequent carcass traits remains limited. Therefore, this study aimed to assess the influence of ambient weather conditions on the correlation between birth body weight (BBW) and carcass characteristics of Korean Hanwoo calves. The BBW and average daily gain (ADG) of Hanwoo male calves vary depending on the month and season of birth, and leading to the hypothesis that exposure to high-temperature environments adversely affects on calf growth and carcass characteristics of hanwoo steers.

## MATERIALS AND METHODS

### Data collection and analysis

Data from 41,107 Hanwoo calves that were collected from Korean local beef farms in 60 cities from 2016 to 2019 were obtained from Dr. Won-Young Lee of the Korea National University of Agriculture and Fisheries. Among them, male calf data containing BBW, BW 90–120 d after birth, and carcass traits of calves slaughtered between 20–40 months of age were selected. The ADG was calculated as:


$$({\text{BW}}_{\text{after}}-{\text{BW}}_{\text{birth}})÷\text{Days},$$

where BW_after_ = BW after 90–120 d of birth, BW_birth_ = BBW, and Days = days between BW_after_ and BW_birth_. Consequently, data from 8,727 Hanwoo male calves (steers) were used for further analyses.

The slaughter age, carcass weight (CW), and meat quality grade were obtained from the Korean Animal Products Traceability System (http://www.mtrace.go.kr). The meat quality grade was calculated after replacing the numbers as follows: 1^++^ grade = 5, 1^+^ grade = 4, 1 grade = 3, 2 grade = 2, and 3 grade = 1. The variables for calf growth and carcass traits were grouped by month and season, as follows: spring (March to May), summer (June to August), fall (September to November), and winter (December to February).

Local ambient temperature and relative humidity (RH) were obtained from the Korea Meteorological Administration. Daily averages of the temperature and RH were used for the analyses. The temperature-humidity index (THI) was calculated according to the NRC [16]:


$$\text{THI}=(1.8\times {\text{T}}_{\text{db}}+32)-\{(0.55-0.0055\times \text{RH})\;(1.8\times {\text{T}}_{\text{db}}-26.8),$$

where T_db_ = dry bulb temperature (°C), and RH = relative humidity (%).

Correlation analyses were conducted between weather variables, BBW, and ADG of the calves and carcass characteristics. The data were grouped and analyzed for the whole period, February and March (FM), and August and September (AS).

IRB/IACUC approval was not required as there were no human nor animal participants.

### Statistical analysis

The data were analyzed using PROC UNIVARIATE in SAS (Enterprise Guide 7.1, SAS Institute Inc., Cary, NC, USA) for the normality test. Normality was determined using the Kolmogorov–Smirnov and Shapiro–Wilk tests. The null hypothesis was accepted because the results of the normality test showed p<0.05; therefore, a non-parametric test was conducted to compare the monthly and seasonal means. All data were analyzed using the Kruskal–Wallis test in the PROC GLM of SAS. The differences between the monthly and seasonal means were analyzed using Dunn’s multiple comparison test, and the p-values were adjusted using the Bonferroni test. Slaughter age was used as a covariate in the CW and meat quality grade analyses. Correlations among climatic conditions, calf growth, and carcass traits were analyzed using the PROC CORR of SAS with the Kendall correlation coefficient. There was no effect of the covariate term, and all data were presented as the least square means±standard deviation. Statistical significance was set at p<0.05.

## RESULTS

### Seasonal patterns of temperature, relative humidity, and temperature-humidity index

**Figure 1:**
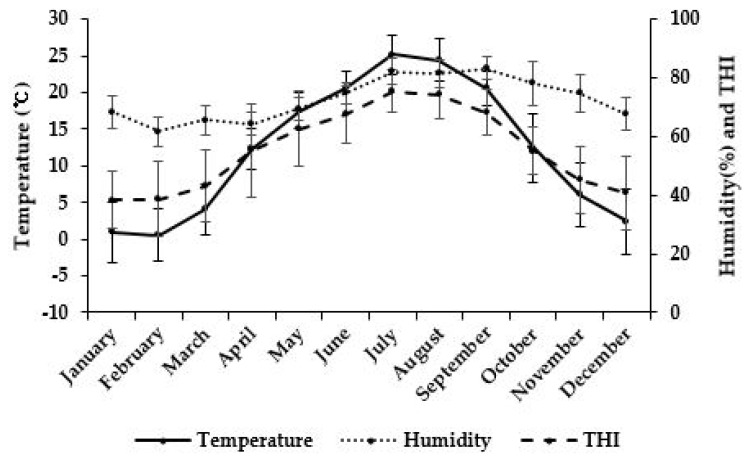
Monthly average ambient temperature, relative humidity, and temperature-humidity index (THI) according to the calf birth month.

The monthly average temperature, RH, and THI showed similar patterns (Figure 1). The monthly average temperatures ranged between 0.55°C and 25.16°C, with low temperature in winter (December to February) and high temperature in summer (June to August) and September. Similarly, the RH was low in winter and high in summer, and THI changes were consequently determined.

### Birth month patterns of birth body weight and average daily gain

**Figure 2:**
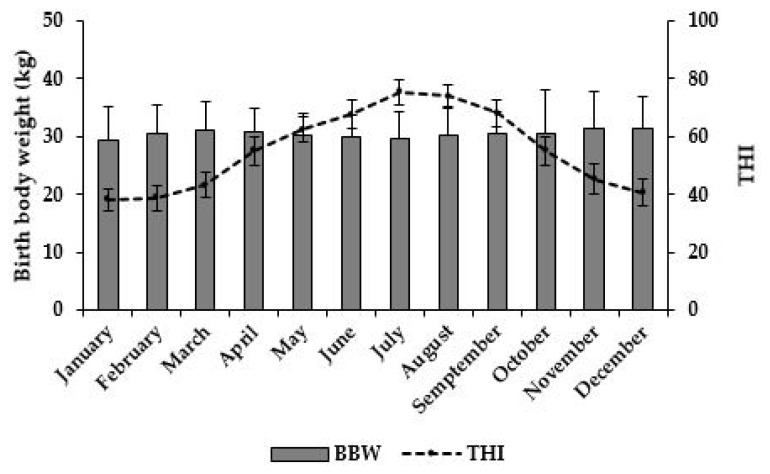
Monthly average birth body weight (BBW) of hanwoo male calves and temperature-humidity index (THI) of the calf birth month.

The number of calves born was highest in August (3,382 calves), followed by February (2,718 calves), March (1,250 calves), and September (919 calves; Table 1). The number of calves born in the remaining months is <200. The BBW of the calves was lowest in January and highest in November (p<0.05; Table 1; Figure 2). Moreover, BBW decreased from March to July, whereas THI increased during the same period. Conversely, the BBW increased from July to November with decreasing THI levels. The ADG at 90- to 120-d after birth was highest in January and lowest in October (p<0.05; Table 1). Monthly ADG decreased from January to May, June to July, and August to October (Table 1).

### Hanwoo steers of slaughter age and meat quality grade

The average slaughter age of all steers was 30.17±1.97 months (Table 2). The monthly average CW decreased from February to May and August to December (p<0.05). The average meat quality grade was 4.00±0.97 (1^+^ grade) and was lowest in December (p<0.05).

### Seasonal patterns of calf birth body weight and steer carcass traits

Calf BBW was significantly lower in summer than in the other seasons (p<0.001; Table 3). The ADG of the calves was lowest in spring (March to May) and fall (September to November), followed by summer (June to August), and was the highest in winter (December to February) (p<0.001). The CW in summer and winter was significantly higher than that in spring (p = 0.001). Hanwoo steers born in winter had higher meat quality grades than spring-born steers (p = 0.025).

### Correlations between climate conditions, growth characteristics, and carcass traits in Hanwoo calves

The ambient temperature, RH, and THI were positively correlated (p<0.01; Table 4). During the period from calf growth to slaughter, climate variables were negatively correlated with BBW and ADG (p<0.01); however, no relationship was observed between climate variables and carcass traits (p>0.10). Positive correlations were observed between BBW, ADG, and CW (p<0.01). The meat quality grade was positively correlated with CW (p<0.01). The FM group presented similar correlation results to the total period group (Table 5). However, there was no relationship between climatic conditions and BBW (p>0.10). Temperature and THI were positively correlated; however, RH showed a slightly negative correlation with both temperature and THI in the AS group (p<0.01; Table 6). Temperature and THI were negatively correlated with BBW and positively correlated with ADG, CW, and meat quality grade in the AS group (p<0.01). The relationships between calf production and carcass traits were similar to those shown in Table 3 and 4, except for the negative relationship between ADG and meat quality grade (p<0.10).

## DISCUSSION

The atmospheric temperature and humidity in Korea exhibit significant seasonal variation, as well as fluctuations in the THI [11]. Figure 1 illustrates combined 4-year data, demonstrating the animals were repeatedly subjected to heat stress.

In Korea, Hanwoo calves are predominantly born during the summer and winter seasons. The calving season varies across countries, influenced by factors such as postpartum performance, feed availability, disease incidence, and climate [17–19]. The seasonal differences in auction prices for Hanwoo cattle may be a potential outcome of these variations. A study analyzing the wholesale price trends of Hanwoo steers throughout the year, the reported price increases in January and September [20]. These increases are attributed to heightened consumer demand for beef during these months, coinciding with traditional holidays in Korea.

The BBW of calves is influenced by various factors, including breed, sex, genetic variance, and the maternal plane of nutrition. Previous studies have reported a decrease in BBW with increasing THI levels in dairy calves [21,22]. This is similar to the results presented in Figure 2 and Table 3. Climatic conditions are a factor affecting BBW as they may influence the nutritional status, stress levels, and immune conditions of calves during the prenatal period [9,23]. When cows are subjected to heat stress, their DMI decreases, reducing the energy required for maintenance and metabolism, which can affect calf birth weight. [7]. This is consistent with a previous study reporting higher DMI during the winter-spring season compared to the summer-autumn season in cows [24]. Therefore, the reduction in BBW during summer (Table 3) is likely attributed to a lower energy state in dams during its transition period. The BBW results for the AS group were negatively correlated with temperature, RH, and THI; however, no such relationships existed in the FM group. This can be explained by monthly THI values. In the present study, the monthly averages of THI were 38.59 to 43.13 in the FM group, indicating that cattle were not stressed by the weather. However, in the AS group, THI decreased from 74.12 to 68.08. Therefore, the observed negative relationship between climatic variables and BBW due to alterations in THI levels from mild heat stress to the threshold condition [25].

The FM and AS groups demonstrated contrasting results regarding the relationship between climatic conditions and ADG of calves. In the present study, ADG was calculated using BBW and weaning weight (BW at 90- to 120-d of birth) indicating that variations in weather conditions from birth to weaning might influence the BW gain of calves. A potential decrease in ADG from spring to summer could be attributed to reduced appetite due to heat stress. This is consistent with the present results, as shown in Tables 1, 3. Therefore, these data influenced the correlation analysis. The birth weight of calves was observed to be lowest in fall, following the summer season. In Korea, fall-born calves have more days of exposure to low temperatures at birth to the weaning period than summer-born calves. According to Korean beef requirements [11], a cold environment reduces weaning weight and ADG after weaning calves. Previous reports have demonstrated inconsistent results for ADG between summer-born and fall-born calves, with a higher ADG in fall-born calves [26] and similar ADG between calves [27]. These varied results appear to be due to temperature differences in the studies’ locations. Another possible explanation is the compensatory growth of August-born calves. It has been reported that compensatory growth following heat stress leads to increased DMI and ADG compared to growth prior to thermal stress in dairy heifer calves [28] and crossbred steers [29]. As the August-born calves underwent hyperthermia, while the September-born ones did not, the former may have exhibited compensatory growth during their recovery period.

According to a previous cohort study on beef cattle, calves with lower BBW exhibited reduced ADG from birth to weaning and lower hot standard CW than the higher BBW calf group [30]. Moreover, a linear regression analysis revealed that a positive correlation between BBW and weight gain in male calves of Holstein, Hereford, and Angus breeds [31]. These findings align with the results of the present study, which revealed positive correlations between BBW, ADG, and CW.

Greenwood [30] suggested that the BBW of calves has minimal impact on meat quality and carcass composition in 30-month-old beef cattle. Similar to our correlation analysis, BBW was positively associated with CW but showed no significant relationship with meat quality grade. However, other studies have reported higher meat quality grades with increased CW in Hanwoo cattle [32,33] and Angus steers [34]. In short, although there was confirmation of relationships between BBW and CW and between CW and meat quality, the correlation between BBW and meat quality remains unconfirmed. Several factors influence marbling scores, including prenatal nutrition, breed, heritability, and animal management [35]. In Korea, consumers treat meat with a high marbling score as high-quality meat; thus, the average slaughter age of beef cattle is approximately 30 months (Table 2). Although there is evidence suggesting a potential link between calf growth and meat quality, the extended raising period and exposure to diverse environmental conditions may have obscured the confirmation of a direct relationship between calf growth and meat quality.

Several studies have investigated CW, meat quality grade and marbling score in Hanwoo cattle based on slaughter season. However, discrepancies in carcass results have been reported across studies. These inconsistencies may stem from differences in the sex composition of the cattle groups used in the studies, such as steers and bulls, or steers, bulls, and cows [36]; and steers, bulls, and cows [37–39]. Moon et al. [32] used only steers in their study; however, their findings differed from those of the present study, likely due to differences in slaughter age (24 months vs. 30 months in the present study). As mentioned earlier, the variability in outdoor environmental conditions between birth and slaughter poses challenges in isolating the effects of weather on cattle performance. Therefore, a long-term investigation is needed to better understand the relationships between Hanwoo cattle performance and climatic conditions at birth, during growth, and at slaughter.

The average monthly THI in the summer season calculated from daily THI values was 67.65–75.48 in this study; indicating that the steers were subjected to heat stress during this period. BBBW exhibited an inverse relationship with weather variables, as evidenced by the negative correlations between BBW and temperature, RH, and THI. The ADG of calves, calculated from birth to weaning, was highest in winter, followed by summer, spring, and fall. CW in Hanwoo steers showed a positive correlation with BBW, calf ADG, and meat quality grades.

Among the many factors influencing the growth traits of calves, the findings of this study suggest potential adverse effects of hot weather on the BBW of Hanwoo male calves, as well as the possibility that calves with higher BBW may achieve greater CW.

## Figures and Tables

**Table 1:** Number of calf births (N), monthly average birth body weight, and monthly average daily gain (mean±standard deviation) of Hanwoo male calves according to the calf birth month

Birth month	N	Birth body weight (kg)	Average daily gain^1)^ (kg/d)
January	18	29.36±3.82^b^	0.80±0.13^a^
February	2,718	30.66±4.26^a^	0.76±0.17^a^
March	1,250	31.05±4.45^a^	0.72±0.18^b^
April	83	30.79±4.86^a^	0.69±0.18^c^
May	161	30.37±4.37^ab^	0.66±0.18^c^
June	38	29.96±5.05^b^	0.71±0.2^bc^
July	25	29.76±4.26^b^	0.64±0.12^c^
August	3,382	30.13±3.88^b^	0.74±0.17^b^
September	919	30.46±4.44^ab^	0.71±0.17^bc^
October	32	30.64±4.96^ab^	0.65±0.14^c^
November	65	31.48±5.02^a^	0.68±0.17^c^
December	36	31.36±4.78^a^	0.67±0.18^c^
Average value		30.49±4.2	0.74±0.17

1) Average daily gain = {(body weight 90–120 days after birth)–(birth body weight)}÷(days between observed body weights).

a–c Within a column, means without a common superscript letter differ significantly, p<0.01.

**Table 2:** Slaughter age (month) and carcass traits (mean±standard deviation) of Hanwoo steers according to the calf birth month

Birth month	Slaughter age (month)	Carcass weight (kg)	Meat quality grade^1)^
January	30.44±2.01	461.39±75.06^ab^	4.17±0.62^a^
February	30.23±1.93	469.93±54.45^a^	4.05±0.94^a^
March	30.02±2.0	464.94±56.38^ab^	4.02±0.95^a^
April	30.16±2.16	458.81±54.0^ab^	3.72±0.99^ab^
May	29.55±2.01	451.37±49.21^b^	3.96±1.04^ab^
June	29.42±2.24	459.45±61.91^ab^	4.11±0.86^a^
July	29.04±1.49	454.76±33.03^ab^	4.04±1.02^a^
August	30.22±1.99	469.31±55.16^a^	3.99±0.99^a^
September	30.2±1.9	466.80±53.65^ab^	3.93±1.03^ab^
October	29.94±1.92	465.31±42.06^ab^	4.19±0.97^a^
November	29.91±1.77	460.77±62.18^ab^	3.78±0.98^ab^
December	29.39±1.48	449.89±43.07^b^	3.42±1.05^b^
Average value	30.17±1.97	467.93±54.92	4.0±0.97

1) 1^++^ grade, 5; 1^+^ grade, 4; 1 grade, 3; 2 grade, 2; 3 grade, 1.

a,b Within a column, means without a common superscript letter differ significantly, p<0.01.

**Table 3:** Number of calf birth, calf growth, and carcass traits (mean±standard deviation) of Hanwoo according to the calf birth season

Variables	Spring	Summer	Fall	Winter	p-value
Number of calf birth	1,494	3,445	1,016	2,772	
Birth body weight (kg)	30.96±4.46^a^	30.13±3.9^b^	30.53±4.5^a^	30.66±4.26^a^	<0.001
Average daily gain^1)^(kg/d)	0.71±0.18^c^	0.74±0.17^b^	0.70±0.17^c^	0.76±0.17^a^	<0.001
Slaughter age (month)	29.98±2.01	30.21±1.99	30.17±1.9	30.22±1.93	
Carcass weight (kg)	463.14±55.65^b^	469.10±55.12^a^	466.37±53.88^ab^	469.62±54.5^a^	0.001
Meat quality grade^2)^	4.00±0.97^ab^	3.99±0.98^ab^	3.93±1.02^b^	4.04±0.94^a^	0.025

1) Average daily gain = {(Body weight after 90–120 d of birth)–(Birth body weight)}÷(Days between observed body weights).

2) 1^++^ grade, 5; 1^+^ grade, 4; 1 grade, 3; 2 grade, 2; 3 grade, 1.

a–c Within a row, means without a common superscript letter significantly differ (p<0.0^1)^.

**Table 4:** Correlations between environmental condition of the calf birth dates, calf growth traits, and carcass traits

Items	Humidity	THI	BBW	ADG^1)^	CW	Meat quality grade^2)^
Temperature (°C)	0.3993**	0.9361**	−0.0368**	−0.0342**	0.0091	−0.0051
Humidity (%)		0.3775**	−0.0293**	−0.0368**	0.0029	0.0008
THI			−0.0391**	−0.0337**	0.0098	−0.0048
BBW (kg)				0.0982**	0.2137**	0.0023
ADG (kg/d)					0.1993**	−0.0077
CW (kg)						0.1524**

1) ADG = {(Body weight after 90–120 d of birth)–(Birth body weight)}÷(Days between observed weights).

2) 1^++^ grade, 5; 1^+^ grade, 4; 1 grade, 3; 2 grade, 2, 3 grade, 1.

** p<0.01.

Temperature, ambient temperature; Humidity, relative humidity; THI, temperature-humidity index; BBW, birth body weight; ADG, average daily gain; CW, carcass weight.

**Table 5:** Correlations between environmental condition of the calf birth dates, calf growth traits, and carcass traits of calves born in February and March

Items	Humidity	THI	BBW	ADG^1)^	CW	Meat quality grade^2)^
Temperature (°C)	0.2091**	0.8027**	0.0161	−0.0667**	−0.0035	0.0036
Humidity (%)		0.0118	0.0177	−0.0220**	−0.0017	0.0136
THI			0.0102	−0.0648**	−0.0009	0.0010
BBW (kg)				0.1009**	0.2309**	0.0037
ADG (kg/d)					0.2103**	0.0099
CW (kg)						0.1370**

1) ADG = {(Body weight after 90–120 d of birth)–(Birth body weight)}÷(Days between observed weights).

2) 1^++^ grade, 5;1^+^ grade, 4; 1 grade, 3; 2 grade, 2; 3 grade, 1.

** p<0.01.

Temperature, ambient temperature; Humidity, relative humidity; THI, temperature-humidity index; BBW, birth body weight; ADG, average daily gain; CW, carcass weight.

**Table 6:** Correlations between environmental condition of the calf birth dates, calf growth traits, and carcass traits of calves born in August and September

Items	Humidity	THI	BBW	ADG^1)^	CW	Meat quality grade^2)^
Temperature (°C)	−0.1488**	0.9175**	−0.0262*	0.0482**	0.0285**	0.0295*
Humidity (%)		−0.0663**	−0.0282**	−0.0202*	−0.0065	0.0169
THI			−0.0304**	0.0478**	0.0283**	0.0330**
BBW (kg)				0.0946**	0.1960**	−0.0082
ADG (kg/d)					0.1881**	−0.0257*
CW (kg)						0.1649**

1) ADG = {(Body weight after 90–120 d of birth)–(Birth body weight)}÷(Days between observed weights).

2) 1^++^ grade, 5; 1^+^ grade, 4; 1 grade, 3; 2 grade, 2, 3 grade, 1.

* p<0.05,

** p<0.01.

Temperature, ambient temperature; Humidity, relative humidity; THI, temperature-humidity index; BBW, birth body weight; ADG, average daily gain; CW, carcass weight.

## References

[1] Korea Meteorological Administration (KMA) (2020). Korean climate change assessment report 2020.

[2] Intergovernmental Panel on Climate Change (IPCC) (2014). Climate change 2013 the physical science basis: working group I contribution to the fifth assessment report of the intergovernmental panel on climate change.

[3] Ataallahi M, Nejad JG, Takahashi J (2019). Effects of environmental changes during different seasons on hair cortisol concentration as a biomarker of chronic stress in Korean native cattle. Int J Agric Biol.

[4] Gregory NG (2010). How climatic changes could affect meat quality. Food Res Int.

[5] Gebregeziabhear E, Ameha N (2015). The effect of stress on productivity of animals: a review. J Biol Agric Healthc.

[6] Sartori R, Sartor-Bergfelt R, Mertens SA, Guenther JN, Parrish JJ, Wiltbank MC (2002). Fertilization and early embryonic development in heifers and lactating cows in summer and lactating and dry cows in winter. J Dairy Sci.

[7] Wang J, Li J, Wang F (2020). Heat stress on calves and heifers: a review. J Anim Sci Biotechnol.

[8] Dahl GE, Tao S, Monteiro APA (2016). Effects of late-gestation heat stress on immunity and performance of calves. J Dairy Sci.

[9] Laporta J, Fabris TF, Skibiel AL (2017). In utero exposure to heat stress during late gestation has prolonged effects on the activity patterns and growth of dairy calves. J Dairy Sci.

[10] Monteiro APA, Tao S, Thompson IMT, Dahl GE (2016). In utero heat stress decreases calf survival and performance through the first lactation. J Dairy Sci.

[11] National Institute of Animals Science (NIAS) (2017). Korean feeding standard for Hanwoo.

[12] Kim WS, Ghassemi Nejad J, Peng DQ, Jo YH, Kim J, Lee HG (2022). Effects of different protein levels on growth performance and stress parameters in beef calves under heat stress. Sci Rep.

[13] Kim WS, Lee JS, Jeon SW (2018). Correlation between blood, physiological and behavioral parameters in beef calves under heat stress. Asian-Australas J Anim Sci.

[14] Baek YC, Kim M, Jeong JY (2019). Effects of short-term acute heat stress on physiological responses and heat shock proteins of Hanwoo steer (Korean cattle). J Anim Reprod Biotechnol.

[15] Baek YC, Choi H, Jeong JY (2020). The impact of short-term acute heat stress on the rumen microbiome of Hanwoo steers. J Anim Sci Technol.

[16] National Research Council (NRC) (1971). A guide to environmental research on animals.

[17] Henry GW, Boyer CN, Griffith AP, Larson J, Smith A, Lewis K (2016). Risk and returns of spring and fall calving for beef cattle in Tennessee. J Agric Appl Econ.

[18] Kim BH, Lee SK, Kim IH, Kang HG (2009). The effect of parity and calving seasons on the reproductive performance of Korean native cows. J Embryo Transfer.

[19] Kwon EG, Cho YM, Park BK, Choi CW, Kim YG, Paek BH (2007). Effect of calving season on growth performance, feed intake and disease occurrence of Hanwoo calves. J Anim Sci Technol.

[20] Choi I, Cho J (2016). Reproduction and marketing plans for improving profitability of Korean native cattle (Hanwoo) farm. J Embryo Transfer.

[21] López E, Mellado M, Martínez AM (2018). Stress-related hormonal alterations, growth and pelleted starter intake in pre-weaning Holstein calves in response to thermal stress. Int J Biometeorol.

[22] Yaylak E, Orhan H, Daşkaya A (2015). Some environmental factors affecting birth weight, weaning weight and daily live weight gain of Holstein calves. Turk J Agric Food Sci Technol.

[23] Almoosavi SMMS, Ghoorchi T, Naserian AA, Ramezanpor SS, Ghaffari MH (2020). Long-term impacts of late-gestation maternal heat stress on growth performance, blood hormones and metabolites of newborn calves independent of maternal reduced feed intake. Domest Anim Endocrinol.

[24] Mazumder MAR, Kumagai H (2006). Analyses of factors affecting dry matter intake of lactating dairy cows. Anim Sci J.

[25] Kim WS, Peng DQ, Jo YH, Nejad JG, Lee HG (2021). Responses of beef calves to long-term heat stress exposure by evaluating growth performance, physiological, blood and behavioral parameters. J Therm Biol.

[26] Broucek J, Kisac P, Uhrincat M (2009). Effect of hot temperatures on the hematological parameters, health and performance of calves. Int J Biometeorol.

[27] Ertugrul O, Alpan O, Unal N, Azeroglu F (2000). Growth and survival of Holstein and Brown Swiss calves reared outdoors in individual hutches. Trop Anim Health Prod.

[28] Appuhamy R, Wickramasinghe J, Stepanchenko N, Oconitrillo MJ, Abeyta M, Goetz B (2021). The effects of diurnal heat stress in dairy Heifer calves. Iowa State Univ Anim Ind Rep.

[29] Mader TL, Davis MS (2004). Effect of management strategies on reducing heat stress of feedlot cattle: feed and water intake. J Anim Sci.

[30] Greenwood PL, Café LM, Hearnshaw H, Hennessy DW, Thompson JM, Morris SG (2006). Long-term consequences of birth weight and growth to weaning on carcass, yield and beef quality characteristics of Piedmontese- and Wagyu-sired cattle. Aust J Exp Agric.

[31] Bailey CB, Mears GJ (1990). Birth weight in calves and its relation to growth rates from birth to weaning and weaning to slaughter. Can J Anim Sci.

[32] Moon SS, Hwang IH, Jin SK, Lee JG, Joo ST, Park GB (2003). Carcass traits determining quality and yield grades of Hanwoo steers. Asian-Australas J Anim Sci.

[33] Park GB, Moon SS, Ko YD (2002). Influence of slaughter weight and sex on yield and quality grades of Hanwoo (Korean native cattle) carcasses. J Anim Sci.

[34] Bruns KW, Pritchard RH, Boggs DL (2004). The relationships among body weight, body composition, and intramuscular fat content in steers. J Anim Sci.

[35] Nguyen DV, Nguyen OC, Malau-Aduli AEO (2021). Main regulatory factors of marbling level in beef cattle. Vet Anim Sci.

[36] Panjono, Kang SM, Lee IS, Lee SK (2009). Carcass characteristics of Hanwoo (Korean cattle) from different sex conditions, raising altitudes and slaughter seasons. Livest Sci.

[37] Kim GW, Kim JH (2017). Analysis of the influence of sex, slaughter season, and feeding system on carcass traits in Hanwoo. Korean J Agric Sci.

[38] Kim YS, Yoon SK, Song YH, Lee SK (2003). Effect of season on color of Hanwoo (Korean native cattle) beef. Meat Sci.

[39] Moon WG, Kim BW, Roh SH (2007). Estimation of environmental effect and genetic parameters for the carcass traits in Hanwoo (Korean cattle). J Anim Sci Technol.

